# Correlative and Dynamic Imaging of the Hatching Biology of *Schistosoma japonicum* from Eggs Prepared by High Pressure Freezing

**DOI:** 10.1371/journal.pntd.0000334

**Published:** 2008-11-11

**Authors:** Malcolm K. Jones, Sze How Bong, Kathryn M. Green, Philadelphia Holmes, Mary Duke, Alex Loukas, Donald P. McManus

**Affiliations:** 1 Queensland Institute of Medical Research, Herston, Queensland, Australia; 2 School of Veterinary Sciences, The University of Queensland, Queensland, Australia; 3 School of Veterinary and Biomedical Sciences, Murdoch University, Perth, Western Australia, Australia; 4 Centre for Microscopy and Microanalysis, The University of Queensland, Queensland, Australia; Swiss Tropical Institute, Switzerland

## Abstract

**Background:**

Schistosome eggs must traverse tissues of the intestine or bladder to escape the human host and further the life cycle. Escape from host tissues is facilitated by secretion of immuno-reactive molecules by eggs and the formation of an intense strong granulomatous response by the host which acts to exclude the egg into gut or bladder lumens. Schistosome eggs hatch on contact with freshwater, but the mechanisms of activation and hatching are poorly understood. In view of the lack of knowledge of the behaviour of egg hatching in schistosomes, we undertook a detailed dynamic and correlative study of the hatching biology of *Schistosoma japonicum.*

**Methodology/Principal Findings:**

Hatching eggs of *S. japonicum* were studied using correlative light and electron microscopy (EM). The hatching behaviour was recorded by video microscopy. EM preparative methods incorporating high pressure freezing and cryo-substitution were used to investigate ultrastructural features of the miracidium and extra-embryonic envelopes in pre-activated and activated eggs, and immediately after eggshell rupture. Lectin cytochemistry was performed on egg tissues to investigate subcellular location of specific carbohydrate groups.

**Conclusions/Significance:**

The hatching of *S. japonicum* eggs is a striking phenomenon, whereby the larva is liberated explosively while still encapsulated within its sub-shell envelopes. The major alterations that occur in the egg during activation are scission of the outer envelope-eggshell boundary, autolysis of the cellular inner envelope, and likely hydration of abundant complex and simple polysaccharides in the lacunal space between the miracidial larva and surrounding envelopes. These observations on hatching provide insight into the dynamic activity of the eggs and the biology of schistosomes within the host.

## Author Summary

Adult schistosomes live within portal veins of their human hosts. Their offspring, laid as eggs within the venous system, escape by traversing the tissues between the blood vessel and the gut or bladder lumen. Eggs voided into the external environment hatch spontaneously on contact with freshwater, and the hatched larva escapes in search of a snail, which acts as intermediate host of the parasite. In this study, we used correlative microscopy techniques to examine hatching of the larvae of *Schistosoma japonicum.* This species has an exquisite hatching behaviour, which allows us to trace the cellular changes in the egg that lead to hatching. By using a correlative microscopy approach, incorporating video microscopy, electron microscopy of eggs prepared by high pressure freezing and lectin immunocytochemistry, we were able to describe the pre-hatching state of the eggs, and trace changes that occur during hatching. The insights gained from these direct biological studies will be of value in understanding the host–parasite interplay of schistosome eggs in their hosts.

## Introduction

The pathology associated with chronic schistosomiasis is related to host responsiveness to antigens released by schistosome eggs entrapped in tissues [1],[2]. The primary source of the secreted antigens in developing eggs is a distinct extra-embryonal layer that surrounds the differentiating embryo (miracidium) [3]. This layer has been variously called the Reynold's layer or the outer envelope (OE), and is derived in early development from cells that delaminate from the embryo [3]–[5]. Secreted antigens, released through preformed pores in the shell [3],[4], consist of a range of peptides and glycans, particularly those containing core mucins and fucose [3],[6],[7].

The schistosome eggshell is a highly cross-linked protein matrix derived from vitelline cells and fashioned in the ootype, an elaboration of the female reproductive system situated between ovary and uterus [8],[9]. Eggshells are formed from precursor proteins, belonging to three families of tyrosine (Tyr)- and glycine-rich molecules [9]. The strong insoluble cross links of the shell are formed by the action of tyrosinases, which catalyze the hydroxylation of Tyr to dihydroxy-phenylalanine, which is subsequently oxidised to dopaquinone for cross linking [10]. An important inorganic component of the shell matrix is iron, which, along with eggshell precursors is stored in the vitelline cells [11]. The shell is largely resistant to degradation by immune effectors in the host or microbes in the environment.

Schistosome eggs are fully embryonated as they escape the host, and as a consequence, miracidia can hatch immediately upon appropriate stimuli in the external environment. The primary cue for miracidial emergence is the osmotic changes that occur as the egg enters freshwater [12]–[14]. Although the physiological and molecular cascades leading to hatching are poorly understood, it has been, hypothesised that they involve, in addition to osmotic changes, calcium fluxes, and the activity of leucine aminopeptidases [3],[13],[14]. In view of the poor knowledge of the biology of egg hatching in schistosomes, we undertook a detailed dynamic and correlative study of hatching biology of *Schistosoma japonicum.* This species, we discovered, has an exquisite and dynamic hatching behaviour. The peculiar nature of its hatching biology enabled us to dissect and understand more clearly the internal cellular changes that occur during the process.

In this study, use was made of the electron microscopy preparative technique of high pressure freezing (HPF). Due to the impervious nature of the trematode eggshell, fixation of its enclosed embryonic structure is notoriously difficult [3],[5] and the resultant ultrastructure of eggs and their content has been poor. An earlier investigation that used the cryo-preparative techniques of slam-freezing in liquid nitrogen to study egg ultrastructure of *S. mansoni* [4], while informative, suffered from artifactual ice crystal damage in the tissues. Similarly, eggs studied after conventional fixation methods of glutaraldehyde fixation were applied [3],[5], suffered from artifact due to slow ingress of fixative into the eggs.

With HPF procedures, samples are snap-frozen in liquid nitrogen at high hydrostatic pressure (approximately 2000 bar) [15]. Under high pressure, water in biological samples is inhibited from nucleating into ice crystals and, thus, is frozen in a vitreous state. Once frozen, samples can be fixed for electron microscopy at low temperature (<−80 C) using a cryo-substitution method which allows fixative to infiltrate samples in the presence of a resin-miscible solvent [15]. Thus, the procedure simultaneously preserves the ultrastructure of the sample and removes water to prevent ice nucleation during thawing. A major benefit of HPF is that specimens can be cryo-immobilized for observations during dynamic activities, methods not possible with conventional preparation methods. Here we describe the dynamic events of hatching of *S. japonicum* egg using HPF to immobilize miracidia for ultrastructural interpretation.

## Materials and Methods

### Preparation of *S. japonicum* eggs

*Schistosoma japonicum* eggs of Chinese mainland (Anhui Province) origin were recovered from livers of infected mice after digestion in collagenase B following described methods [16]. Eggs were stored for 2 days in Petri dishes in sterile PBS at 4°C until use. The use of mice in this study was approved under Project P288 by the Animal Ethics Committee of the Queensland Institute of Medical Research.

To study hatching behaviour of larvae, eggs were transferred to aged distilled water. Real-time video recordings were made at 100× magnification using a Panasonic Color CCTV Camera (Model WV-CP610/G), connected to the video output of a Zeiss inverted microscope.

### Ultrastructure of S. *japonicum* eggs during hatching

Eggs and miracidia were fixed for transmission electron microscopy at three stages of the hatching process, as follows:

- Pre-activation.
- 1.5 h after transfer of eggs into distilled water at room temperature, at which time, approximately 50% of larvae were activated and many had hatched. Other eggs were exposed to 10 µM praziquantel for 10 min in phosphate buffered saline (PBS), diluted from a stock solution in dimethyl sulphoxide (DMSO) to induce spontaneous hatching [17].
- Immediately after rupture of the eggshell.

Eggs from stages A and B were transferred to a solution of 20% (w/v) bovine serum albumen in PBS on a proprietary copper membrane and rapidly frozen in a Leica EM PACT2 High Pressure Freezer (Leica, Wetzlar, Germany). Subsequently, the membranes and samples were transferred in cryo-tubes under liquid nitrogen to a Leica EM AFS freeze substitution apparatus for fixation and dehydration in 2% (w/v) osmium tetroxide and 0.5% uranyl acetate (w/v) in 100% anhydrous acetone. The tissues were cryo-substituted for 3 days, according to the following protocol. The temperature of the substitution chamber was increased from −160°C to −85°C over 2 h, and maintained at −85C for 48 h, after which the samples were brought to room temperature. The osmium-uranyl acetate-acetone solution was then replaced with anhydrous acetone at room temperature. After further changes of acetone, the samples were infiltrated with Epon resin (ProSciTech, Townsville Australia). Final infiltration of resin was facilitated in a Pelco 34700 Biowave Microwave Oven (Ted Pella Inc., Redding, USA).

Eggs from stage C were prepared by conventional EM processing as the miracidia had escaped the eggshell and were thus more easily fixed. For this, liver-derived eggs were incubated at room temperature in aged water in 24-well plates. As soon as a larva and its enveloping structures were observed to break from the shell, an aliquot of 3% glutaraldehyde (v/v) in 0.1 M phosphate buffer, pH7.4 was introduced into the well, thus immediately fixing and immobilizing the miracidia. The fixed miracidia were postfixed in 1% osmium tetroxide, then dehydrated in acetone at room temperature and embedded in Epon resin.

### Electron Microscopy

Ultrathin sections were cut at 60 nm, mounted onto uncoated or formvar/carbon coated copper grids, and stained with uranyl acetate and Reynold's lead citrate. The sections were examined in a JEM1011 transmission electron microscope operated at 80 KV and photographed using a digital camera. EM observations were made from multiple specimens from each sample. Hatching experiments in water and praziquantel were repeated three times.

### Lectin Cytochemistry

Ultrathin sections of high pressure frozen and freeze substituted pre-activated eggs (stage A), were subjected to lectin cytochemistry using biotinylated Concanavalin A, Wheat Germ Agglutinin, Peanut Agglutinin and *Ulex Europaeus* Agglutinin (Vector Labs, Burlingame, USA). The carbohydrate specificity of the lectins is published [18],[19] and presented here also in Table 1. All lectins were diluted to 10 µg/ml in HEPES buffer, using 0.5% (w/v) gelatin as the blocking agent. Subsequently sections were labelled with rabbit anti-biotin antiserum (Bethyl Laboratories, Montgomery, USA) diluted 1∶200 in gelatin/PBS followed by protein-A conjugated to 10 nm gold particles (Utrecht University, Netherlands) in the same buffer. Sections were stained with uranyl acetate and lead citrate, and viewed in a JEM1011 transmission electron microscope as described above.

**Table 1:** Carbohydrate distribution in eggs of unactivated eggs of *S. japonicum,* demonstrated by ultrastructural lectin cytochemisty on resin sections.

Lectin	Specificity	Shell	Pores	Reynolds Layer	Inner Envelope	Rosettes	Lipoid Bodies	Miracidium		
								Tegument	Terebratorium	Penetration Glands
**ConA**	Α-D-Mannose>α–D-glucose>N-acetyl-α–D-glucosamine	A		A	P lysosomes	P	P	P septate desmosomes	A	P
**UEA**	α-L-Fucose	A	P	A	A	A	A	A	A	A
**WGA**	N-Acetyl-β-glucosamine	A	P	A	A	A	P	A	A	A
**PNA**	β-D-galactosamine	A	P	A	A	A	P	A	P	P

The letter “P” indicated presence of label over structures, whereas “A” indicates no or negligible label over that structure. Corresponding labelling patterns of the lectins are shown in Fig. 7. Abbreviations: ConA- Concanavalin A (*Canavalia ensiformis*, Jack bean), UEA- *Ulex europaeus* agglutinin I, WGA- Wheat germ agglutinin, PNA- peanut agglutinin.

## Results

### Hatching Behaviour of *S. japonicum* Eggs

**Figure 1:**
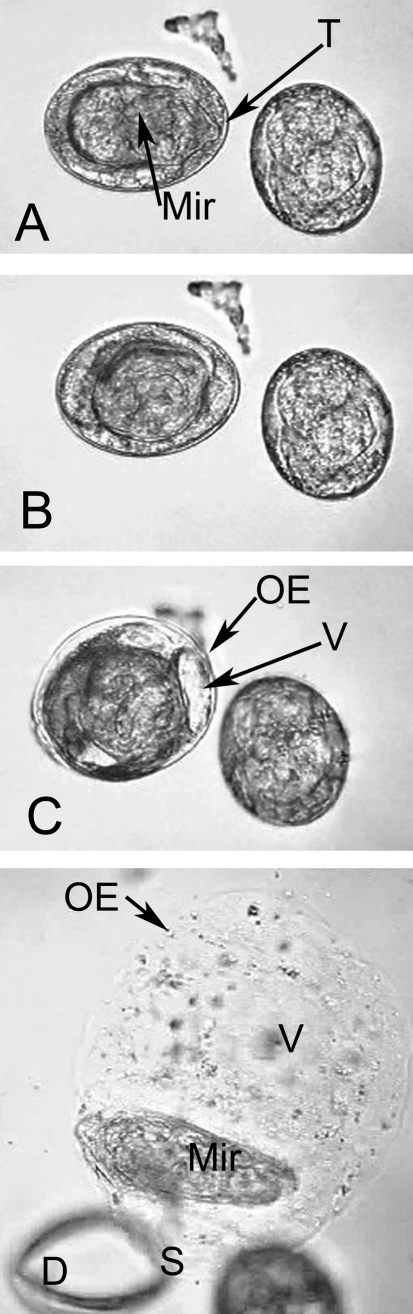
Hatching of the egg of *S. japonicum,* light microscopy. A. Activated larva prior to translocation in the shell. The egg on the left hand side contains an activated larva. Here the miracidium lies longitudinally in the egg and its terebratorium is clearly evident. B. The same larva has turned so that it lies across the egg. C. Egg immediately after shell rupture. The outer envelope and vacuoles are swelling and obscure the now smaller shell. D. The outer envelope is now markedly expanded and the miracidium swims actively within its increasingly viscous matrix. The shell lies to the bottom left of the figure, and is slightly out of focus. Abbreviations: OE- outer envelope; Mir- miracidium; S-shell; T-terebratorium.

The hatching behaviour is shown in Videos S1, S2, S3 and S4 and Fig. 1. Another version of the hatching biology in a movie (made by MKJ) has been deposited in the TDR website of the World Health Organization with the title *The Great Escape* (currently file is at: http://www.who.int/tdr).

After exposure to water, most miracidia do not display activity within the egg shells for at least twenty minutes, although some hatched spontaneously. Larvae continued to activate and hatch over a 24 hour period.

Evidence of activation included arrhythmic twitching of the miracidium and rapid ciliary beating (Videos S1 and S2). These activities continued for a variable and sometimes prolonged period of time. The contents of the matrix within the shell became progressively clarified and large vacuoles in the lacuna surrounding the miracidium become more apparent. In most miracidia (Video S3, and “*The Great Escape”*), shell rupture occurred only after the miracidium swung its body from a longitudinal to a lateral orientation within the shell, so that the long axis was perpendicular to the shortest axis of the egg. This change in orientation did not occur for the miracidium filmed in Video S1, but the egg in this specimen appeared smaller than surrounding eggs, suggesting that it is traction against the shell walls that was important rather than orientation, for this and other miracidia appear to use muscular activity to aid in shell rupture. The shell always ruptured along the long axis of the egg (see fracture plane in empty shells in the background of Videos S1, S2, S3 and S4).

Following shell rupture, the larva emerged, enclosed within a sac formed by an extra embryonic “membrane”, the outer envelope (OE;  =  Reynold's layer [4]). The OE underwent rapid expansion until it filled a volume at least twice that of the shell (Videos S1, S3). Vacuoles within the OE also expanded at similar rates and continue to expand after the larva has escaped the OE (Video S1). The miracidium swam rapidly and erratically within the sac, stopping on occasions to push its anterior organ, the terebratorium, against the limiting OE. The medium in which the miracidium swam became increasingly viscous as the larva circled within it. Eventually, the miracidium stopped and forced its way through the OE, thereby liberating itself and the viscous contents of the sac.

### Ultrastructure of Non-activated Eggs

As assessed by fixation quality of internal structure of the egg, particularly mitochondria and endoplasmic reticulum, membranes, and the absence of ice crystal damage, the use of HPF produced superior ultrastructure of encased miracidia and investing envelopes. Different authors have used a variety of names to describe the ultrastructure of the schistosome egg [3]–[5]. The result is a confusing nomenclature. The system proposed by Swiderski [5], which uses names commonly used for egg structures in the Platyhelminthes [20], is adopted here, although alternative nomenclature is also given.

#### Shell

**Figure 2:**
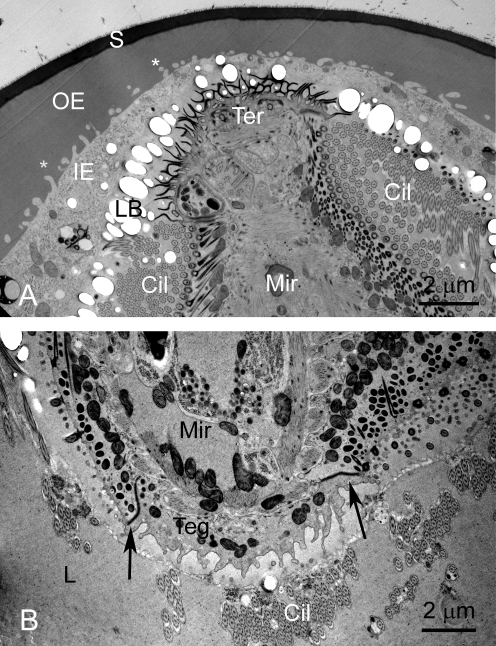
Eggs of *S. japonicum*, shown by TEM, prepared by HPF and cryo-substitution. A. Anterior region of a miracidium, showing the terebratorium. The miracidium is enveloped by, in turn, the lacuna, containing lipoid bodies, the inner and outer envelopes, and the shell. Inpocketings of the outer envelope (asterisk) occur at the boundary of the inner and outer envelopes. B. Posterior end of the miracidium in the lacuna. Note the septate desmosomes (arrows) separating the epithelial plates of the miracidial tegument. Abbreviations: Cil- cilia; IE- inner envelope; L- lacuna; LB- lipoid bodies; Mir- miracidium; S- shell; Ter- terebratorium.

**Figure 3 pntd-0000334-g003:**
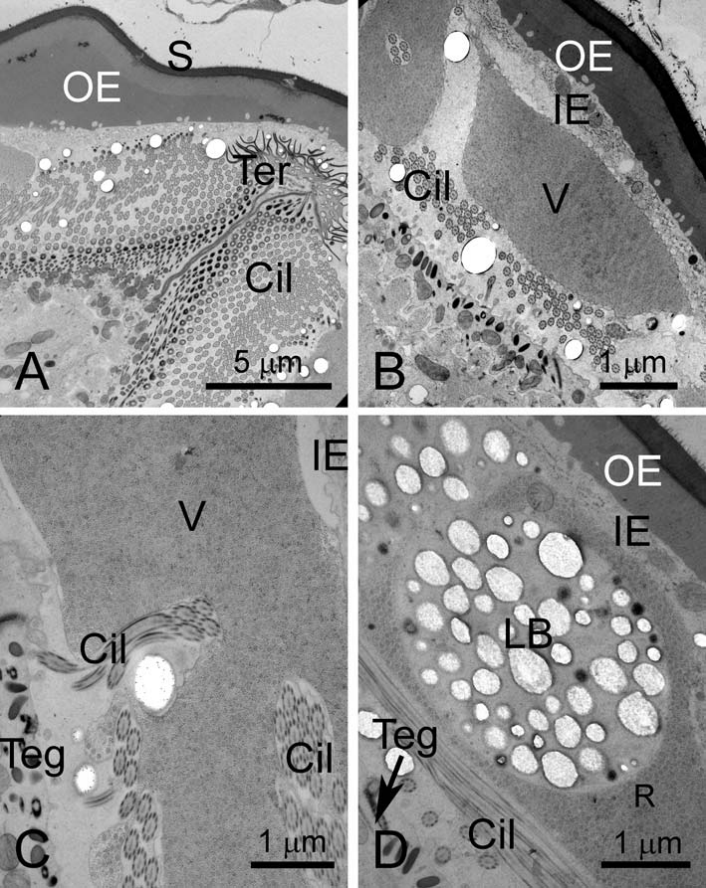
Eggs of *S. japonicum*, shown by TEM, prepared by HPF and cryo-substitution. A. Anterior region showing terebratorium of miracidium. B. Vacuole in lacuna of egg. C. Higher magnification of a vacuole. D. Aggregration of lipoid bodies in a vacuole. Abbreviations: Cil- cilia; IE- inner envelope; L- lacuna; LB- lipoid bodies; Mir- miracidium; S- shell; Teg- tegument; Ter- terebratorium; V-vacuole.

**Figure 4:**
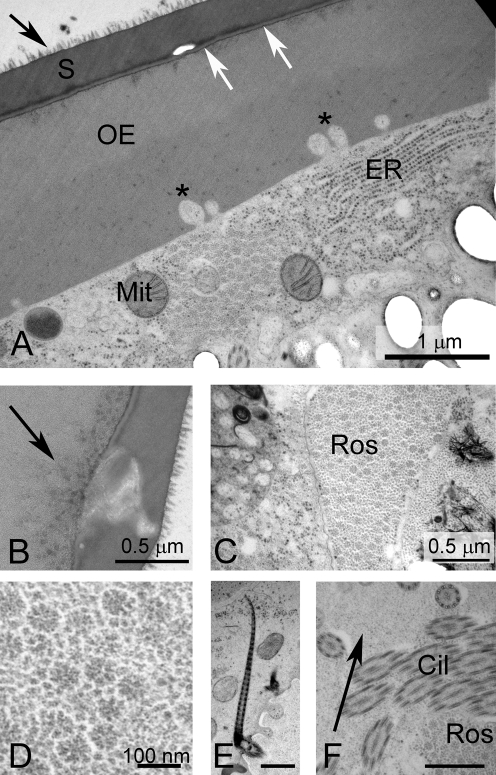
Eggs of *S. japonicum*, shown by TEM, prepared by HPF and cryo-substitution. A. Inner and outer envelope. Note the boundary between the shell and outer envelope (arrows). B. Pore in shell. Aggregations of a granular material lie in the outer envelope just beneath the pore (arrows). C. Rosettes in a vacuole. D. High magnification view of rosettes. E. Ciliary rootlet of miracidial tegument. Scale bar  =  1 micron. F. High magnification of lacuna. The matrix of the lacuna is granular Scale bar  =  0.5 microns.

The shell (Figs 2–4) has an electron opaque matrix with two prominent layers, an outer region consisting of microspines (Figs 3A, 3B, 4B) [3]–[5]) and an inner amorphous region that forms the shell proper (Fig. 2A, Fig. 3A, 4A). The shell has a uniform appearance and is of uniform thickness. The shell matrix is interrupted by pores containing a relatively electron-lucent material and is lined by linear structures (Fig. 4B). Underlying the shell is a thin lamina, consisting of electron-lucent material sandwiched between electron-opaque membrane-like structures (Fig. 3A–B).

#### Extra-embryonic Envelopes

The OE is a thick layer immediately subjacent to the shell (Figs 2A, 3A–B, 4A–B). This layer has a densely fibrous matrix, composed of a dense meshwork of interlocking filaments. Small spherical particles form aggregates beneath the shell pores (Fig 4B). The OE is underlain by the cellular inner envelope (IE) [5] ( = von Lichtenberg's layer [4]; Figs 2A, 3A–B,D, 4A). The IE is a thin cellular layer with variable thickness. At its margin with the IE, the OE displays abundant invaginations into which the cytoplasm and membrane of the IE extend (Figs 2A, 4A).

The IE has multiple nuclei, but no intercellular boundaries are evident, suggesting that this layer is syncytial. The cytoplasm is rich in rough endoplasmic reticulum, lysosomes and mitochondria (Figs 2A, 3A–B,D, 4A). Additionally, an abundant feature of the cytoplasm is the accumulated rosette bodies (Fig. 4A), reminiscent of α-glycogen rosettes (Fig. 3D). The rosettes are packaged into membrane-bound vacuoles [14] ( = Cheevers bodies [4]) and exported from the cell. The vacuoles appear as dominant components of the lacuna between the miracidium and inner envelope (Figs 3B–C, 4C). Rosettes also lie free in the lacuna (Fig. 4F). Aggregations of electron-lucent lipoid bodies [4], that are limited externally by a membrane-like boundary, are present within a cytoplasmic matrix of the IE (Fig 3D).

#### Lacuna

The miracidium lies within a lacunal space immediately subjacent to the inner envelope. The matrix of the lacuna is a moderately electron-opaque granular substance. Inclusions within this extra-embryonic lacuna include the large vacuoles filled with rosettes (Figs 3 C–D), as well as a material resembling β-glycogen granules (Figs 4E–F). Lipoid bodies [4] are particularly abundant around the anterior extremity of the miracidium (Figs 2A, 4A).

#### Miracidium

The miracidium has a cellular tegumentary epithelium, consisting of ciliated epithelial blocks delimited by septate junctions (Fig. 2B). Mitochondria are abundant at the base of the tegument. The cilia (Fig 2A–B; 3A, C, 4E–F) have a typical 9+2 arrangement of microtubules with deep ciliary rootlets (Figs 2A, 3E). The cilia intermesh with the other constituents of the lacuna, and often appear entrapped within vacuoles (Fig. 3C). The miracidium has a distinct terebratorium [21],[22] at its anterior extremity (Fig. 2A). The membrane of the terebratorium is highly villous, is lined externally with an electron opaque material, and lacks cilia. Cilia are also lacking from the epithelial plate that lines the posterior extremity of the parasite (Fig. 2B).

### Dynamic Imaging of *S. japonicum* Eggs During Hatching

Rapid freezing of samples in HPF ensures the simultaneous immobilization of all components of tissues, and is thus optimal for providing a snapshot of dynamic activity in cells and tissues [23]. We activated eggs in distilled water to induce hatching and immobilised eggs after 1 h to observe the structural changes that occurred in the membranes during hatching. Because individual miracidia respond to osmotic changes at different time-points and, thus hatch at different times throughout a 24 h time period, it is difficult to prepare multiple specimens by HPF. For this reason, eggs of *S. japonicum* were exposed to praziquantel to induce rapid and near synchronous hatching of all mature eggs [17]. There are some deleterious effects of praziquantel administration on miracidia. The major effect is on the musculature of the miracidium, which contracts markedly, so that the larvae appear pinched in their midline. The miracidia can still swim, however, through active motility of their cilia. Miracidia still move their bodies to lie laterally across the egg prior to shell rupture. A second effect is that some components of the lacuna do not separate completely from the cilia, so that some hatched larvae may drag sub-shell components through the aquatic milieu.

**Figure 5:**
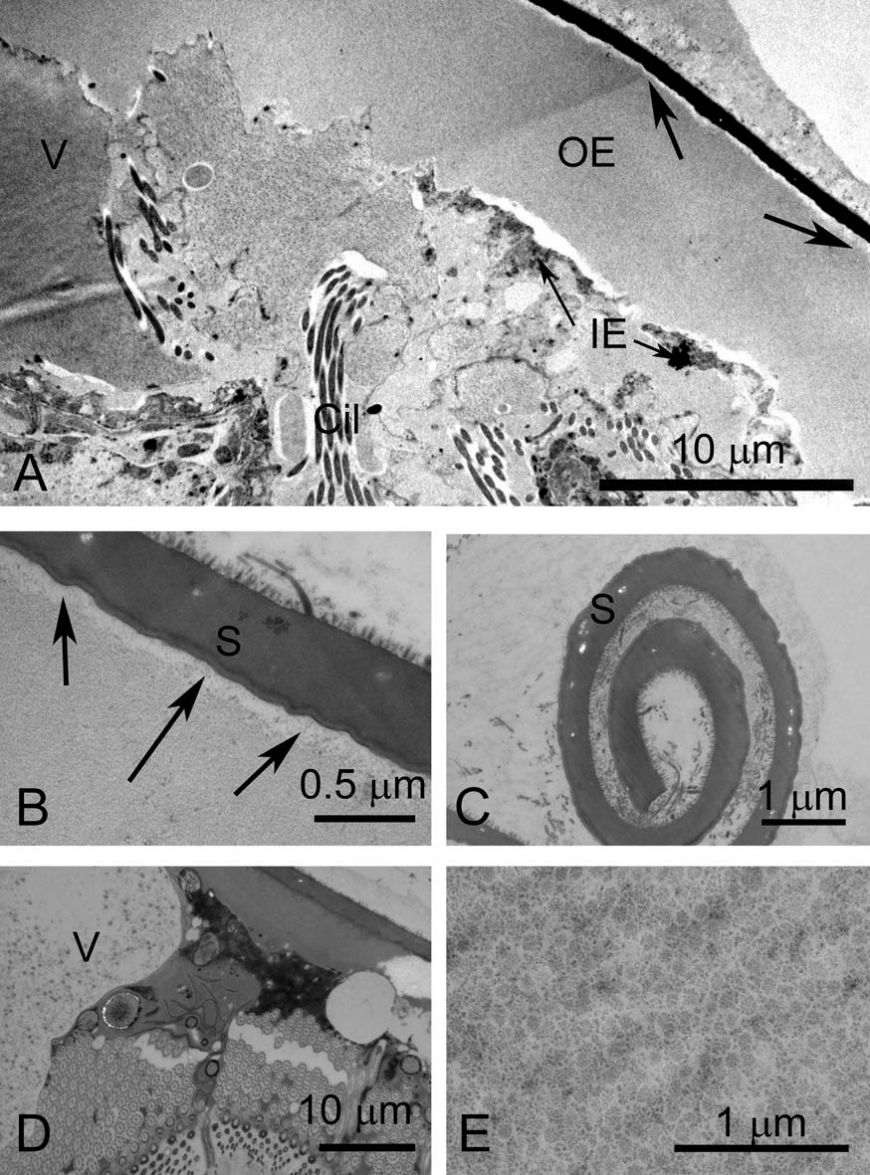
Eggs of *S. japonicum* induced to hatch in water or praziquantel, shown by TEM, prepared by HPF and cryo-substitution. A. Praziquantel-treated egg. Note the separation of the shell and the outer envelope (arrows). The inner envelope is partially degraded. B. Praziquantel-treated egg, showing separation of the shell from the outer envelope (arrows). C. Shell of an egg hatched in water, at the site of fracture. D. Egg hatched in water. A vacuole with mostly degraded rosettes is shown. E. Egg hatched in water, showing partially degraded rosettes. Abbreviations: IE- inner envelope; OE- outer envelope; S- shell; V-vacuoles.

Four changes were obvious in eggs that had been induced to hatch by praziquantel or water. Firstly, the basal lamina of the shell separated from the OE so that a distinct space was observed between the two envelopes (Fig 5A–B). In shells ruptured by hatching, the shell material appeared completely separated from the OE (Fig. 5C). Secondly, the lacuna surrounding the miracidium lost its flocculo-granular matrix and the matrix became clearer. Thirdly, lipoid bodies became distributed throughout the lacuna and were not aggregated anteriorly. Fourthly, the rosettes within the membrane sacs became less distinct and showed evidence of degradation (Figs 5D, E).

### Ultrastructure of Embryonic Envelopes Immediately After Shell Rupture

**Figure 6:**
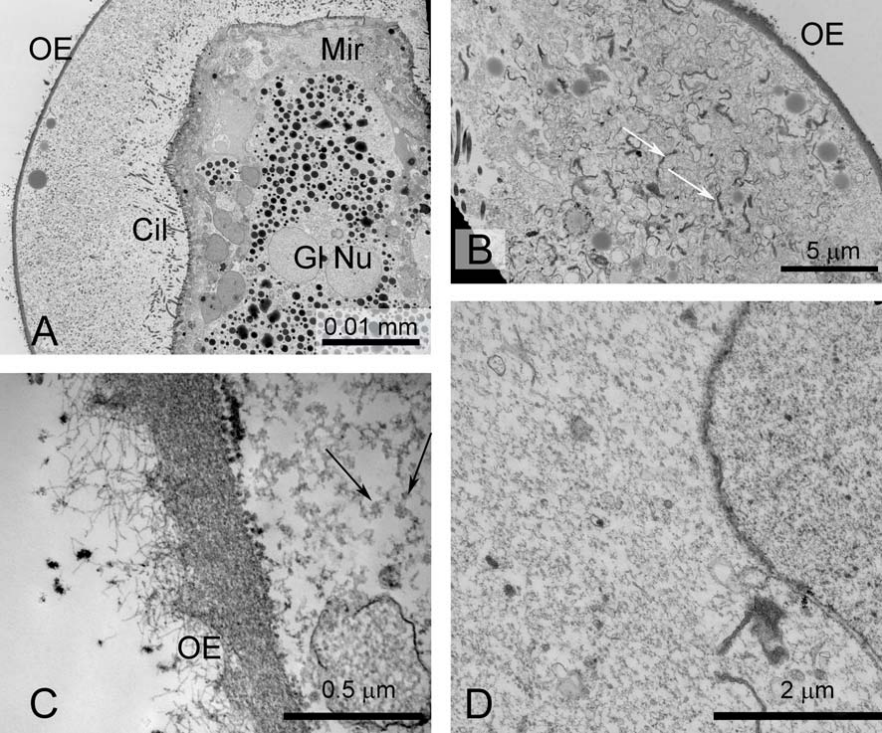
Miracidia of *S. japonicum*, fixed immediately after shell rupture for conventional processing for TEM. A. Low magnification image showing miracidium within the expanded lacuna. The penetration glands are evident in the miracidium. B. Expanded lacuna with numerous membranes (arrows). C. Outer envelope. Note that the external fibrils appear to be unravelling. Partly degraded rosettes are shown (arrows). D. Lacuna, showing membrane body, presumably a vacuole. Cil- cilia; Gl Nu- nuclei of penetration glands; Mir- miracidium; OE- outer envelope.

Immediately after eggshell rupture, the OE and vacuoles expanded rapidly (Videos S1, S3). When miracidia and attendant envelopes were fixed during this phase of expansion, the only ultrastructurally identifiable features were the OE and the miracidium (Figs 6A–D). The OE remains as an electron-opaque fibrous layer, although it was much thinner than in it was in the pre-activatedn egg (Figs 6A–C). The branched fibrils of the OE separated from each other at the external water interface (Fig. 5C). Other contents of the egg, including IE, were degraded and replaced by a coarsely granular material and numerous membrane profiles, some of which were reminiscent of endoplasmic reticulum. The vacuoles were represented by membranes surrounding a granular substance (Fig. 5A–C).

### Lectin Cytochemistry of Non-activated Eggs (Fig. 7; Table 1)

**Figure 7:**
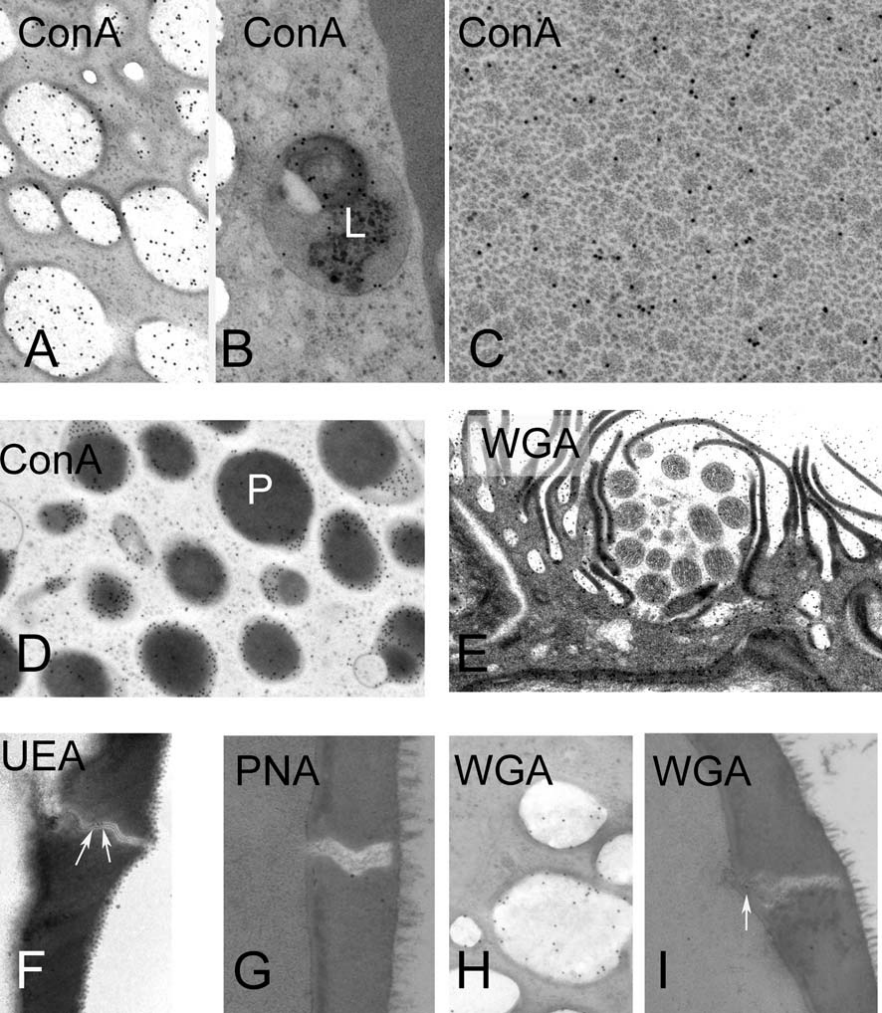
Lectin cytochemistry of *S. japonicum eggs,* shown by TEM, prepared by HPF and cryo-substitution. The panels show representative regions with positive localization with the lectin. A–D Con A cytochemistry. A. Lipoid bodies B. Lysosome in inner envelope. C. Rosettes in vacuoles; D. Penetration gland of miracidium. E. WGA labelling in lacuna surrounding the terebratorium. F. UEA in pore of shell. G. PNA in pore of shell. H. WGA in lipoid body. I. WGA in pore.

Lectin cytochemistry of the eggs (Fig. 7; Table 1) revealed a variable distribution of carbohydrate moieties throughout the egg shell constituents. No lectin bound to egg shell, although there was weak lectin binding for UAE, WGA and PNA in the pores. The OE stained weakly with ConA. Con A labelled a range of structures, but particularly, the rosettes, matrix of the lacuna, regions of the miracidial tegument and vesicles of the penetration glands, lipoid bodies and lysosomes of the IE (Fig. 7; Table 1). PNA reactivity was observed only at the periphery of secretory bodies of the penetration glands and at the periphery of lipoid bodies. WGA reactivity was detected only within the lipoid bodies.

## Discussion

This study, the first detailed study of the ultrastructure of *S. japonicum* eggs, shows that the use of HPF as a means of immobilizing the eggs and egg contents provided a superior method of specimen preparation compared with other methods used previously for studies of *S. mansoni* eggs [3]–[5]. The structure of the eggshell and its sub-shell compartments were preserved with high quality and without freezing artifacts. Despite the use of osmium tetroxide as fixative, some cytochemical labelling using biotinylated lectins in conjunction with colloidal gold probes was possible, as has been demonstrated for studies on insects and nematodes [24]. The ability of the technique to immobilize eggs during hatching, allowing morphological and cytochemical observations, helps overcome problems brought about by the impervious shell [25]. The method provides a means of adding valuable data on the biological roles of drugs, such as praziquantel.

Based on the vital, structural and cytochemical studies described here, the following conclusions can be made about the components of the *S. japonicum* egg. The shell is a dense homogenous layer and is completely devoid of carbohydrate residues. Numerous microspines are present at the surface and, along with the terminal egg spine, may provide traction for the egg to adhere to vascular endothelium in the mammalian host. For *S. mansoni,* the interaction of eggs and endothelium is intimate, as evidenced by the rapidity with which the host tissues overgrow freshly deposited eggs [26].

The shell itself is a dense homogenous layer, interrupted in places by cribriform pores. In all mature eggs observed here, the shell was of uniform density and thickness. Interestingly, our videos of hatching *S. japonicum* miracidia showed that the shell always ruptures along the long axis of the shell, indicating the presence of a pre-formed weakness in the shell along this axis. Such a line of weakness could arise from a different chemistry in certain regions of the shell, from the occurrence of a thinner band of eggshell material at the line of weakness, or by accumulation of pores in that area. None of these, however, were apparent in this study.

The outer envelope of the schistosome egg consists of a filamentous material [4], composed of numerous interlocked fibrillar proteins. The layer is closely applied to the eggshell, and likely bonds with the latter structure. The junction between the two layers was clearly broken in water- and praziquantel-induced hatching. After shell rupture, the OE thinned considerably as the mass it enclosed expanded rapidly. The filaments of the OE also appeared to unravel in the presence of water, suggesting that cross-links between filaments are water-soluble or readily hydrated.

Secreted antigens produced by the egg must traverse the OE to escape the egg. Many secreted antigens are thought to arise in the IE [3] and there is substantial evidence that secreted antigens are generated by mature schistosome eggs [3],[7], as well as immature eggs, which lack the fibrous OE. Apart from the observation of small particles in regions of the OE subjacent to pores, there is little morphological or cytochemical evidence of antigens traversing the OE. The carbohydrate fucose, along with mucin-rich compounds, are abundant components of glycoproteins secreted by *S. mansoni* eggs [6], but cytochemical analysis here did not reveal abundant carbohydrate components in the OE. Nevertheless, the mature OE is likely to be a water soluble matrix, as evidenced by the seemingly rapid transit of water through the layer and its behaviour in freshwater.

Ashton and colleagues have proposed that the inner envelope is the primary source of secreted antigens in *S. mansoni* eggs [3]. The cellular layer is highly synthetic, and observations here indicate that among its possibly many functions can be listed the remodelling of the OE, secretion of proteins, and generation of substantial carbohydrate components present in the rosettes and lipoid bodies. Swiderski [5] described acid phosphatase reactivity in the IE of *S. mansoni* eggs just prior to hatching, and suggested that autolysis of this layer provided substantial nutritional benefit to the miracidium. In *S. japonicum* eggs, the IE degrades only after activation, suggesting that the IE remains extant prior to activation to maintain extraembryonic components of the egg.

Structures in the lacuna of mature *S. japonicum* eggs bind lectins indicating the presence of carbohydrate moieties. Rosettes stain with ConA, which indiscriminately binds glucose and mannose. Similar structures in the related digeneans *Fasciola hepatica* and *Echinostoma caproni* also contain carbohydrate and are involved expansion of the viscous cushion, a structure that leads to rupture of the opercular boundary in their eggs [4],[5],[19]. The so-called lipoid bodies are also rich in carbohydrates, particularly glucose, mannose, galactosamine and N-acetyl-glucosamine, as is the general matrix of the lacuna.

The process of egg hatching appears similar in *S. japonicum* and *S. mansoni* [13],[14]. One of the clear differences between the two species, however, is that for *S. japonicum,* the OE emerges to form a sac around the escaping miracidium. Based on the accumulated evidence from a range of studies [13],[14],[17],[19],[27], the following model of egg hatching can be postulated for *S. japonicum.*

On contact with freshwater, osmosis induces the inflow of water into the egg, probably through the pores. Kusel [14] noted that *S. mansoni* eggs swell prior to hatching, indicating that the water inflow is abundant. Osmotic pressure appears to be a major factor in hatching of schistosome eggs [13]. In the related schistosome,*Trichobilharzia regenti*, a parasite of birds, it appears that other factors stimulate hatching [28]. In that species, larvae hatch within the nasal tissues of their avian hosts, stimulated by light or by slight osmotic change with inflow of water into the nasal cavities. The influence of other environmental parameters on hatching of eggs of *Schistosoma* species, especially light, is uncertain, as miracidia hatch in the dark [13].

Praziquantel, as noted here and by others [17],[29] induces rapid hatching of schistosomes eggs in the absence of an osmotic gradient. There is mounting evidence that praziquantel modulates calcium transport in schistosomes [30],[31]. Praziquantel causes hatching in PBS, implying that its effect is independent of osmotic pressure and likely involves the flux of calcium into the tissues of the eggs. Whether this modulation of calcium levels occurs in the inner envelope or miracidium to induce hatching remains to be determined.

One of the immediate outcomes of egg activation in *S. mansoni* is the rapid release of the hydrolytic enzyme leucine aminopeptidase (LAP), which has been recorded in hatching fluids upon release of miracidia from eggs [13],[32]. Administration of praziquantel to *S. mansoni* eggs also induces an immediate release of LAP [29]. Blocking of LAP activity in eggs with bestatin, a general inhibitor of aminopeptidases, effectively blocks hatching activity [13],[32]. LAPs are found in the gastrodermis and tegument of adult schistosomes [33], but the site of expression in the eggs is unknown. One likely source of the enzyme is the IE itself, which in *S. japonicum* possesses lysosomes, and in *S. mansoni* contains compartments which stain for acid phosphatase, a marker of acidic compartments where LAPs are likely to function [5]. LAPs are terminal enzymes in catalytic cascades, liberating amino acids, particularly N-terminal leucines from peptides [33],[34]. The natural substrate of LAP from eggs is uncertain, but potential roles for the enzyme include scission of the OE-shell boundary, autolysis of the IE itself, or perhaps to assist in degradation of proteins that occur in the carbohydrate-laden lacunae.

Data on egg hatching in the parasitic Platyhelminthes is scant, but for those species that have been studied it appears that many use a form of extra-embryonic viscous cushion or vacuole that expands to induce pressure increases within the egg (reviewed in [13],[19],[35]). It has been calculated that a pressure of 35 megaPascals (5000 psi) is required to rupture schistosome eggshells [13]. Schmidt [19] used lectin cytochemistry in light microscopy studies to demonstrate that glycogen-like carbohydrate components were abundant in vesicles in the egg lacunae of *F. hepatica* and *E. caproni*. Schmidt argued that while polymerised forms of polysaccharides are generally water insoluble, their depolymerization will lead to greater water solubility and water binding potential. The lacuna of *S. japonicum* eggs appears to be enriched for carbohydrate polymers. Moreover, the potential for rapid expansion of the OE sac once liberated from the ruptured shell suggests a rapid osmosis-driven influx of water, mediated perhaps by the unravelling of previously hydrophobic molecules. The absence of rosettes and lipoid bodies in hatched eggs (Fig. 6) suggest that these complex polysaccharides do, indeed, depolymerise.

Based on the new information described here, we postulate that the eggs of schistosomes, and particularly, *S. japonicum,* proceeds as follows. Eggs exist within the mammalian host under isosmotic conditions. When transferred to freshwater, an inflow of water signals calcium fluxes in the external membranes of the IE of the miracidium, leading to activation of potentiating enzymes that initiate scission of the OE-eggshell boundary, autolysis of the IE and partial depolymerization of polysaccharides in the lacuna. The potential involvement of LAP in this process remains undetermined. With the dissolution of structures, and aided by mechanical activity of the miracidial cilia, the intra-ovular environment becomes free allowing for partial motility of the miracidium so that it can change its orientation in the egg. The increased pressure afforded by the expanding polysaccharides, together with muscular activity of the larva itself forces rupture of the shell. The freed OE allows the sudden influx of water, a flow previously limited by the physical constraints of the pores, and the OE sac expands rapidly. With more space to move, the miracidium swims rapidly, but stops intermittently to force a break in the OE, through which it finally escapes.

It remains to explain the selective advantage of the swelling OE to ensure hatching and overall continuation of the life cycle of *S. japonicum.* The answer possibly and most simply is that the OE serves to push out a clear space in the faecal mass containing the egg to give the larva an opportunity to escape into surrounding water.

## Supporting Information

Video S1 Hatching biology of *S. japonicum* miracidia. The video commences after activation of the larvae by freshwater. Although 2 larvae are activated, only one hatches in the time frame of the video. Note that the split in the eggshell is longitudinal, and this larva doesn't rotate through 90 degrees prior to shell rupture.
(5.23 MB WMV)

Video S2 An activated *S. japonicum* egg prior to shell rupture.
(1.73 MB WMV)

Video S3 Shell rupture of a *S. japonicum* egg. After 24 sec into the video, the miracidium rotates within the shell, leading to rupture.
(2.99 MB WMV)

Video S4 A miracidium emerging from a cracked shell. The outer envelope does not separate fully from the shell, and the larva re-enters the shell prior to escape.
(2.21 MB WMV)
